# Development of surface modified bilosomes for the oral delivery of quercetin: optimization, characterization in-vitro antioxidant, antimicrobial, and cytotoxicity study

**DOI:** 10.1080/10717544.2022.2122634

**Published:** 2022-09-18

**Authors:** Nabil K Alruwaili, Ameeduzzafar Zafar, Omar Awad Alsaidan, Mohd Yasir, Ehab M. Mostafa, Sultan F. Alnomasy, Alenazy Rawaf, Ali Alquraini, Fadhel A. Alomar

**Affiliations:** a Department of Pharmaceutics, College of Pharmacy, Jouf University, Sakaka, Al-Jouf, Saudi Arabia; b Department of Pharmacy, College of Health Sciences, Arsi University, Asella, Ethiopia; c Department of Pharmacognosy, College of Pharmacy, Jouf University, Sakaka, Al-Jouf, Saudi Arabia; d Department of Medical Laboratories Sciences, College of Applied Medical Sciences in Al-Quwayiyah, Shaqra University, Shaqraa, Saudi Arabia; e Department of Medical Laboratory, College of Applied Medical Sciences-Shaqra, Shaqra University, Shaqraa, Saudi Arabia; f Department of Pharmaceutical Chemistry, Faculty of Clinical Pharmacy, Al Baha University, Al Baha, Saudi Arabia; g Department of Pharmacology and Toxicology, College of Clinical Pharmacy, Imam Abdulrahman Bin Faisal University, Dammam, Saudi Arabia

**Keywords:** Quercetin, bilosomes, chitosan, antioxidant, breast cancer

## Abstract

Quercetin (QT) is a flavonoid that exhibits anti-oxidant and chemo-preventive activity. This research work aimed to develop surface-modified bilosomes (BS) of QT. The BS was prepared by the solvent evaporation method and optimized by the Box-Behnken design. The optimized QT-BS (QT-BS3opt) displayed vesicle size (143.51 nm), PDI (0.256), zeta potential (−15.4 mV), and entrapment efficiency (89.52%). Further, the optimized QT-BS formulation was coated with chitosan (CS). The XRD diffractogram of CS-QT-BS3opt1 did not exhibit extensive peaks of QT, revealing that QT is properly encapsulated in the polymer matrix. The QT-BS3opt and CS-QT-BS3opt1 exhibited sustained-release (86.62 ± 3.23% and 69.32 ± 2.57%, respectively) up to 24 h with the Korsmeyer-Peppas kinetic model (R^2 ^=0.9089). CS-QT-BS3opt1 exhibited significantly (*P* < .05) high flux, i.e. 4.20-fold more than pure QT dispersion and 1.27-fold higher than QT-BS3opt. CS-QT-BS3opt1 showed significantly greater bio-adhesion (76.43 ± 2.42%) than QT-BS3opt (20.82 ± 1.45%). The antioxidant activity showed that QT from CS-QT-BS3opt1 has more remarkable (*P* < .05) antioxidant activity at each concentration than pure QT. The CS-QT-BS3opt1 exhibited 1.61-fold higher cytotoxicity against MFC7 and 1.44-fold higher cytotoxicity against MDA-MB-231 than pure QT. The CS-QT-BS3opt1 displayed a significantly greater antimicrobial potential against *E. coli* than against *S. aureus*. From all these findings, it could be concluded that surface-modified QT-BS might be an effective approach for increasing the efficacy of QT in the treatment of certain ailments.

## Introduction

Quercetin (QT) is an herbal natural bioactive compound belonging to the polyphenol flavonoid category. It is obtained from various sources like citrus fruit, vegetable, and beverages (Anand David et al., 2016). In vegetable sources, the most abundant are Allium cepa and other medicinal plants like Hypericum perforatum and Ginkgo biloba (Kim and Kim 2006; Yang et al., 2020). There are various pharmacological activities of QT reported by researchers, such as antioxidant (Lesjak et al., 2018), antiviral (Johari et al., 2012), antimicrobial activity (Streptococcus aureus; Escherichia coli; Helicobacter pyroli; Yersinia enterocolitica, etc.) (Wang et al., 2018), anti-inflammatory (Li et al., 2016), hepatoprotective and antihypertensive (Lekić et al., 2013). Alzheimer’s disease (Brown et al., 2005) and anti-cancer activity (Rauf et al., 2018). Its pharmacological activity mostly takes place through active phenolic hydroxyl groups and double bonds. QT has antimicrobial activity by inhibiting cell membranes (Wang et al., 2018); antioxidant activity by scavenging reactive oxygen species (Boots et al., 2008); and anticancer activity by directly inducing apoptosis in cancer cells (Shafabakhsh and Asemi, 2019). Despite its pharmacological characteristics, ‘QT is a poorly water-soluble (0.00215 g/L at 25 °C) (Srinivas et al., 2010) with low bioavailability (4%), poor permeability, gastrointestinal instability, and low absorption in the gastrointestinal tract, which limits its biological effects in-vivo (Reinboth et al., 2010; Souza et al., 2014). Due to these limitations, QT has poor therapeutic activity. Various nano-formulation approaches for improving the therapeutic efficacy of poorly soluble therapeutics have been reported, such as pegylated polylactic co-glycolic acid nanoparticles of Curcumin and Chrysin (Lotfi-Attari et al., 2017), nanostructured lipid carriers (NLCs) of Artemisinin (Emami et al., 2018), liposome delivery of dihydroartemisinin and glycyrrhetinic acid (Kang et al., 2017; Tian et al., 2014), solid lipid nanoparticle (SLN) of Silibinin (Xu et al., 2013), and BS delivery of curcumin (Abbas et al., 2022). Various formulation approaches have been reported by researchers for the enhancement of the therapeutic efficacy of QT. Patel and his associates reported the liposome delivery of QT for breast cancer (Patel et al., 2020). It displayed a 1.94-fold increase in half-life and a significant increment in in-vivo anticancer activity (rats). Varshosaz and his associates reported the SLN delivery of QT for liver cancer (Varshosaz et al., 2014). It showed significantly lower IC_50_ (2-times) than pure QT and significantly higher accumulated liver malignant cells (HepG2 cells in vitro). Li and his associates formulated QT-SLN with a 571.4% enhanced relative bioavailability as compared to pure QT (Li et al., 2009). Sun and his associates formulated the NLCs of QT. It had a particle size of 32 nm, 95% entrapment efficiency, 11% drug loading, and a 1000-fold increase in QT solubility (Sun et al., 2014). Another researcher formulated the polymeric micelle of QT for the treatment of lung cancer. It significantly increases the cytotoxicity against A549 cancer cells and is 2.5 times lower in IC_50_ than pure QT (Zhi-Yong et al., 2017). It is stated that the therapeutic activity of flavonoids can be improved by incorporating them into the colloidal system. Various studies have reported the application of lipid-based formulations for improving the solubility, stability, and therapeutic activity of poorly soluble therapeutics because they increase the effective surface area of absorption. Many lipid-based vesicular formulations like noisome, liposome, transfersome, BS, etc., have recently been used by researchers as drug delivery carriers for the improvement of therapeutic effects (Abbas et al., 2022). Nowadays, BS are widely used as drug carriers because they have good intestinal stability and deformability. It is a modified noisome having bile salt (sodium deoxycholate or sodium glycocholate), lipid, cholesterol (CHO), and surfactant. Bile salt improves the stability of the lipid bilayer (Abbas et al., 2022) and the biological stability of several drugs like acyclovir (Saifi et al., 2020), curcumin (Abbas et al., 2022), tetanus toxoid (Mann et al., 2006), vaccines (Wilkhu et al., 2013), etc. Cholesterol enhances the membrane rigidity of the bilayer and increases the encapsulation efficiency (Abbas et al., 2022). Coating of BS with chitosan (CS) further may improve the in-vitro and in-vivo effectiveness of the therapeutics. CS is a natural biological macromolecular polymer with biocompatibility, biodegradability, and bio-adhesive properties. It also has antimicrobial activity by adsorption of amino groups on the cell bacteria and leading to leakage of the cellular constituents. It enhanced intestinal permeation by opening the tight junction (Zhou et al., 2018). It is a cationic macromolecule that interacts with anion molecules to form the protective layer and increases cell permeability (Cuomo et al., 2018).

The current research project entails the development of the QT-BS using the solvent evaporation method and optimizing it with the Box-Behnken design. The QT-BS was assessed for vesicle size and entrapment efficiency. Further, the optimized QT-BS was coated with different concentrations of CS and evaluated for in-vitro release and ex-vivo egg permeation. Furthermore, the optimized CS-QT-BS was envaulted for in-vitro biological activity, i.e. in-vitro antioxidant, in-vitro cytotoxicity, and antimicrobial activity.

## Material and method

### Material

Quercetin, Chitosan (deacetylation degree ≥ 95%, viscosity 100–200 mpa.s), chloroform, methanol, and acetonitrile were procured from Sigma Aldrich (Bengaluru, India). Span 60 and sodium deoxycholate (SDC) were acquired from SD fine Chemicals (Mumbai, India). 1,1-diphenyl-2-picrylhydrazyl (DPPH) and ABTH were obtained from Sigma Chemical Co. (St. Louis, MO, USA). The rest of the chemicals utilized in this experiment are of analytical grade.

### Methods

#### Optimization by experimental design

The quercetin (QT) loaded BS formulations were optimized using 3-factors and 3-levels Box-Behnken experimental design software (Design Expert 8.0.6, Stat-Ease, Minneapolis, Minnesota, United States). The independent factors were lipid, span-60, and SDS, while the dependent variables were vesicle size (VS, nm), entrapment efficiency (EE, %), and polydispersity index (PDI). Table 1 shows the results of a total of 13 formulations with various components. To determine the best fit model, the responses were fitted into various experimental models, including linear, second-order, and quadratic models. All of the employed models were statistically analyzed. An ANOVA was performed and a polynomial equation was generated for the best-fitted model of each response. The response surface (3D and contour) was constructed, which expressed the effect of formulation factors over the responses individually and in combination. The observation of the relationship between the actual and predicted value graphs of each response was constructed to find closeness between them. Furthermore, point prediction of the software was used to obtain the optimized formulation.

**Table 1.** Formulation composition of quercetin-loaded liposome and value of responses (VS, EE, and PDI).

Formulation code	Lipid (A, %)	Span-60 (B, %)	SDC (C, %)	Vesicle size (nm)	EE (%)	PDI
QT-BS1	4	3	8	244.21	61.24	0.41
QT-BS2	6	3	8	346.34	77.66	0.467
QT-BS3	4	7	8	143.51	89.52	0.256
QT-BS 4	6	7	8	192.21	93.21	0.348
QT-BS 5	4	5	4	190.87	76.62	0.349
QT-BS 6	6	5	4	272.84	88.65	0.432
QT-BS 7	4	5	12	219.16	72.41	0.363
QT-BS 8	6	5	12	287.13	83.01	0.43
QT-BS 9	5	3	4	262.18	74.12	0.459
QT-BS 10	5	7	4	180.45	91.13	0.323
QT-BS 11	5	3	12	330.51	65.21	0.463
QT-BS 12	5	7	12	157.81	90.06	0.327
QT-BS 13	5	5	8	221.45	81.54	0.358

#### Development of quercetin-loaded bilosomes

The QT-loaded BS (QT-BS) was developed by the solvent evaporation method with a slight modification of the previously reported method (Waglewska et al., 2020). The required amounts of QT, lipid, span-60, and SDC were weighted (Table 1) and dissolved inorganic solvent (chloroform: methanol, 1:1). The aqueous solution of bile salt (SDC) was prepared separately and heated up to 45○C. The organic phases were added dropwise into an aqueous phase with continuous stirring. The organic solvent evaporated and a BS vesicle was formed and stood for 2 h with stirring. The developed QT-BS was sonicated for 5 min in a bath sonicator (Optics Technology, India). The QT-BS was stored at a cool temperature for further evaluation. For evaluation, the developed QT-BS was kept at a cold temperature.

#### Characterization of QT-loaded bilosomes M_0_^1/3 ^−M_t_^1/3 ^=Kt (Hixon-Crowell)

A zeta sizer (Malvern zeta-sizer, Malvern, UK) was used to determine the size, PDI, and zeta potential of developed QT-BS vesicles. The QT-BS was diluted twenty times using milli-Q water and measured for VS and PDI at a 90° scattering angle. For zeta potential, the same diluted sample was filled into an electrode cuvette and subjected to the measurement of zeta potential.

#### Entrapment efficiency determination

The ultracentrifugation method was employed for the estimation of EE. The QT-BS was placed in a centrifugation tube and centrifuged at 15,000 rpm (Remi, India) for 30 min. The supernatant was collected and diluted appropriately. Absorbance was measured by UV-Vis-spectrophotometry (Shimadzu 1800, Japan) at 370 nm. The given Eq. (1) was used to compute the percent EE.

(1) $$\%\;EE=\frac{\mathrm{Total}\;QT\;\mathrm{used}-QT\;in\;\mathrm{supernatant}}{\mathrm{Total}\;QT\;\mathrm{used}}\times 100\;\ldots .$$

#### Development of CS-coated QT-BS

The optimized QT-BS formulation was coated with CS. The required quantity of CS (0.20–0.30%) was weighed and dissolved in 0.1% v/v of aqueous acetic acid solution (Abo El-Enin et al., 2022). The CS solution was added to the optimized QT-BS formulation under continuous stirring for 3 h at room temperature.

#### Vesicle size, PDI, and zeta potential of CS-coated QT-BS

The VS, PDI, and zeta potential of the CS-coated QT-BS (CS-QT-BS) was determined by the zeta sizer (Malvern zeta sizer Malvern, UK).

#### X-ray diffraction study

The X-ray diffractogram of QT, CHO, SDC, QT-BS, CS, and CS-QT-BSopt was captured by the X-ray diffraction instrument (Rigaku International Corporation, Japan). Each sample was placed individually into a sample holder, converted into a thin film, and placed into an instrument. The image was captured at 2 theta level after scanning the sample at 5^○^–40^○^. The instrument was operated at 40.0 kV voltage and 30 mA current, with a scanning speed of 2.0 deg/min.

#### In-vitro release study

The dialysis bag method was used to evaluate in-vitro drug release of QT from QT-dispersion, QT-BS, and CS-QT-BS-dispersion. To open the pores, the dialysis bag was immersed overnight in distilled water. The release medium (phosphate buffer, pH 6.8, 100 ml) was poured into a beaker and maintained at 37 ± 0.5 ○C using a thermostat magnetic stirrer. The 1 ml of QT-dispersion, QT-BS, and CS-QT-BS dispersion (equivalent to 5 mg of QT) were filled into a dialysis bag and bound tightly. After that, the dialysis bag was immersed in a release medium and constantly stirred at 100 rpm. At a predetermined time point, 3 ml of the sample was taken from the beaker and added to the same volume of fresh media in the beaker to maintain a constant volume. The release sample was filtered with a membrane filter (0.25 m) and the concentration of QT was determined using the previously reported HPLC method (Abdelkawy et al., 2017). The mobile phase consists of 0.25% aqueous trichloroacetic acid and acetonitrile (45:55) and a flow rate of 0.75 ml/min.

#### Kinetic release study

The release data of the optimized CS-QT-BS-dispersion was fitted into various kinetic models i.e. zero order, first order, Higuchi model, Korsmeyer-Peppas, and Hixon-Crowell. The equation of all models is given below.

(2) $$Q_{t}=Q_{0}-{\;k}_{0}t\;\left(\text{zero}\;\text{order}\right)$$

(3) $${\text{logQ}}_{t}={\;\text{logQ}}_{0}-k_{1}t/2.303\;\left(\text{First}\;\text{order}\right)$$

(4) $$\text{Qt}={\;k}_{H}t^{1/2}\left(\text{Higuchi}\right)$$

(5) $$\text{Mt}/M\infty \;={\text{kt}}^{n}\left(\text{Korsmeyer}-\text{Peppas}\right)$$

(6) $$M_{0}{}^{1/3}-M_{t}{}^{1/3}=\text{Kt}\;(\text{Hixon}-\text{Crowell})$$

In that Q_t_ = drug release over time t; Q_0_ = amount of drug at zero time; K_0_ = zero order constant; K_1_ = first order rate constant; K_H_t (Higuchi constant); M_t_ & M_∞_ = amount of drug release at time t and infinitive time; M_0_ = amount drug at zero time; M_t_ = amount of drug remaining at time t; k = Korsmeyer-Peppas an dHixon-Crowell constant; n = diffusion exponent for releases mechanism; *n* = 0.45 indicated Case1 or fickian diffusion; 0.45 < *n* < 0.89 = anomalous behavior or non-fickian diffusion; *n* = 0.89 (Case 11 transport) and *n* > 0.89 = super case II transport (Pudjiastuti et al., 2020).

#### Bio-adhesive study

A bio-adhesive study optimized QT-BS and CS-QT-BS was carried out by employing pig mucin. The 1 mg/ml of mucin solution was prepared in double-distilled water. The QT, QT-BS, and CS-QT-BS were mixed with mucin solution (1:1) and stood for 4 h at room temperature. After finishing the incubation period, the sample was centrifuged at 15,000 rpm for 30 min, and the supernatant was isolated. Free mucin concentration in the supernatant was observed by UV-spectrophotometry at 251 nm (Papadimitriou et al., 2008). The % bio-adhesion was calculated by the given Eq. (7).

(7) $$\%\;\mathrm{Bioadhesion}=\frac{\mathrm{Total}\;\mathrm{mucin}\;\mathrm{amount}\;\mathrm{used}-\mathrm{free}\;\mathrm{mucin}\;in\;\mathrm{supernatant}}{\mathrm{Total}\;\mathrm{mucin}\;\mathrm{amount}\;\mathrm{used}}\times 100.$$

#### Ex-vivo permeation

The egg membrane was used for permeation of QT from QT-BS and CS-QT-BS because the egg membrane exhibits similarity to the intestinal membrane (Haigh and smith 1994). A diffusion cell was used in the experiment. The egg membrane was detached from the egg by immersing it in 1 N HCl and then cleaned. The phosphate buffer (pH 6.8, 20 ml) was taken into the acceptor compartment of the diffusion cell as permeation media and kept at 37 ± 0.5○C with constant stirring (100 rpm). The egg membrane was placed between both compartments and 0.5 ml of QT, QT-BS, and CS-QT-BS dispersion (2 mg of QT) was filled into the donor compartment. The 2 ml of the permeation media were taken and added to the same volume of fresh buffer to maintain a constant volume. The sample was filtered and diluted, and the content was analyzed by HPLC (Abdelkawy et al., 2017). The flux and apparent permeability coefficient (APC) were calculated. The APC was calculated by the following Eq. (8):

(8) $$\mathrm{APC}=\frac{\mathrm{Flux}}{\mathrm{Area}\times \text{initial QT concentration}}$$

### In-vitro anti-oxidant study

#### DPPH radical scavenging method

*In-vitro* antioxidant activity of QT and CS-QT-BS was measured by the DPPH free radical scavenging method (Clarke et al., 2013). The various concentrations of QT and CS-QT-BS (10–150 µg/ml) from stock solution (1 mg/ml) were prepared in methanol. The 0.1 M of DPPH was prepared in methanol and stored at 4^○^C until used. For the completion of the reaction, 500 µL of each sample was mixed with 100 µL of DPPH solution and stood for 1 h in a dark place. When the reaction was completed, the color changed to colorless from violet, indicating the scavenging activity. The absorbance was measured by UV-spectrophotometry at 571 nm. A blank (butylated hydroxytoluene, BTH) was taken as a control. The percent antioxidant activity of the tested sample was calculated by the following Eq. (9).

(9) $$\text{\% Antioxidant activtiy}=\frac{\text{Abs of control}-\text{Abs of test sample}}{\text{Abs of control}}\times 100$$

#### ABTS radical scavenging method

The study was performed as per the previously described method with a small modification (Chaves et al., 2020). The various concentrations of QT and optimized CS-QT-BS (10–150 µg/ml) were prepared and 0.1 ml of each dispersion was mixed with 3.9 ml of ABTS solution and vortexed. The mixture was incubated for 30 min in a dark place and analyzed by UV-Vis spectrophotometry at 734 nm. The BTH solution is taken as a blank for comparative analysis. The percentage of scavenging activity was calculated by the following formula 10 (Loganayaki et al., 2013).

(10) $$\text{\% Antioxidant activtiy}=\frac{\text{Abs of control}-\text{Abs of test sample}}{\text{Abs of control}}\times 100$$

#### Cytotoxicity study

The MTT assay method was used to test the cytotoxicity of pure QT and optimized CS-QT-BS on breast cancer cell lines (MCF-7 and MDA-MB-231). The cell line was obtained from an American culture type (ATCC, Manassas, USA). MCF-7 and MDA-MB-231 cells were cultured in Dulbecco’s Modified Eagle Medium supplemented with 10% fetal bovine serum, 100 U/ml streptomycin, and 100 U/ml penicillin. The cell was grown at 37 °C/95% RH with a continuous supply of 5% CO_2_ (Galaxy® 170 R CO_2_ incubator, Eppendorf, Germany) and maintained a pH of 7.4 (Kim et al., 2011; Sun et al., 2014). The stock solution tested samples (QT & CS-QT-BS) and was diluted with phosphate buffer to avoid toxicity. The MCF-7 and MDA-MB-231 cells (5 × 10^3^) were seeded into a 96-well sterile microplate and allowed to stand overnight. The cells in the 96-well plate were treated with each concentration of QT and CS-QT-BS for 48 h. 0.05 mg/ml of MTT was filled into each well of the 96-well plate and incubated at 37^○^C for 2 h with a continuous supply of 5% CO_2_ and 95% O_2_. After that, DMSO (100 µL) was added to a 96-well plate to dissolve the formazan crystal. Finally, a microplate reader was utilized to evaluate the mixture, with blank DMSO serving as a control. The IC_50_ and the percentage of cells inhibited were calculated by the given Eq. (11).

(11) $$\text{\% Cell inhibition}=\;\frac{\text{OD of control}-\text{OD of test sample }}{\text{OD of control}}\;\times 100$$

#### Antimicrobial study

The antimicrobial evaluation of pure QT-dispersion and CS-QT-BS was done on *Staphylococcus aureus* (*S. aureus*, gram-positive) and *Escherichia coli* (*E. coli*, gram-negative) microbial strains using the cub plate method. The culture was grown in nutrient broth media equivalent to 5 × 10^6^ CFU/ml bacterial load. The nutrient agar medium was prepared and sterilized at 121^○^C in an autoclave. The 0.5 ml (diluted) of microbial strain was mixed with liquid nutrient agar media and transferred to a sterile Petri plate under aseptic conditions and allowed to stand for solidification. After solidification, the 6 mm cup was made using a sterile stainless-steel borer. The test sample (QT and CS-QT-BS, 100 µL) was filled into each cup and stood for 2 h for absorption of the test sample. Then a Petri plate was placed into the incubator in an inverted position at 37^○^C for 24 h. The zone of inhibition (ZOI) was then measured using a graduated scale.

#### Statistical analysis

Experimental design software (version 8.0.6) was used for the optimization of the formulation. The value was represented in mean ± SD. GraphPad (InStat, CA, USA) was used for statistical analysis. One-way ANOVA and Tukey-Karman multiple comparison tests were used for data analysis. Significant differences were taken at *P* < .05.

## Result and discussion

### Optimization

**Figure 1.**
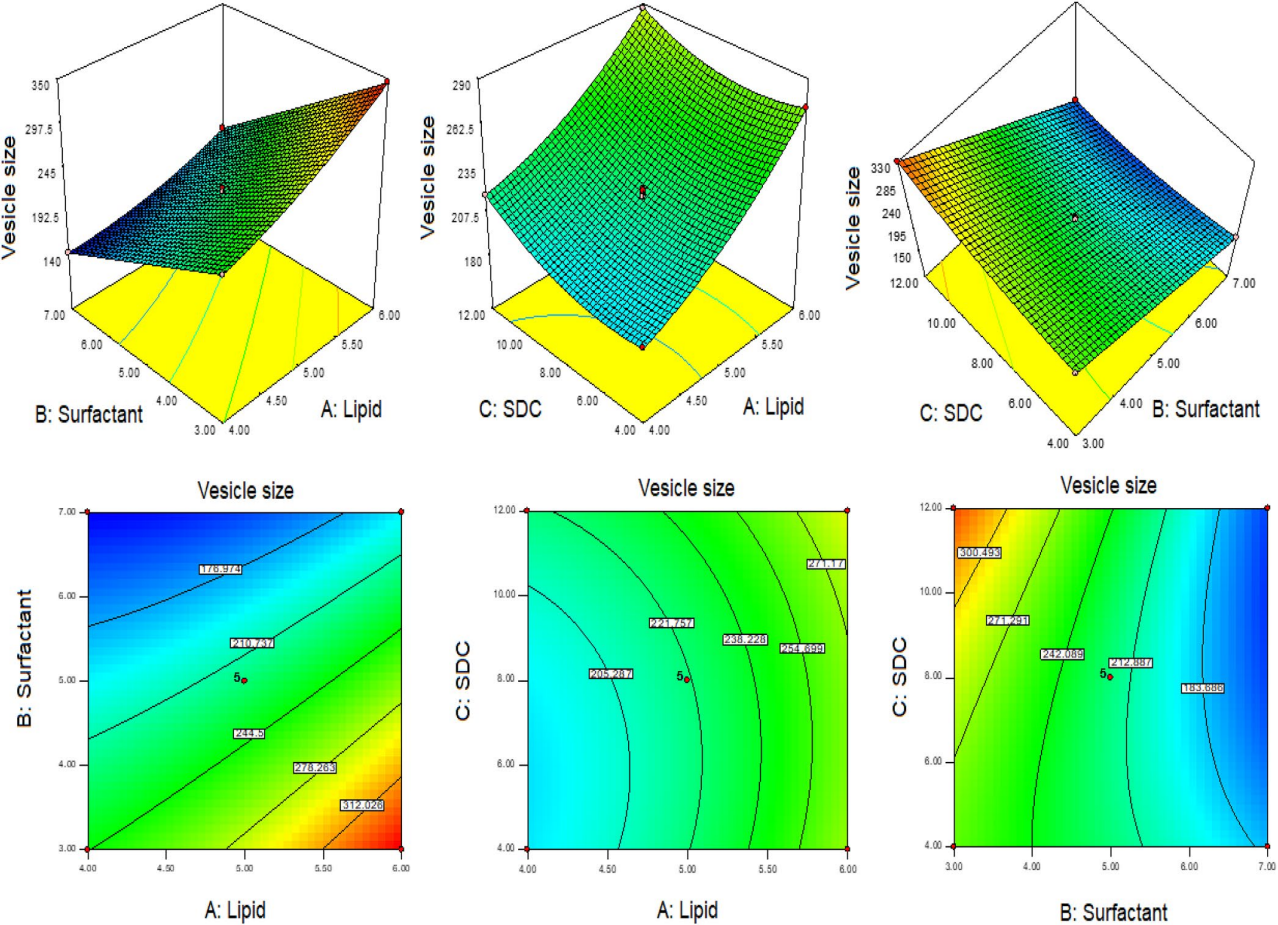
3D and contour plots showing the effect of independent variables on the particle size of QT-BS.

**Figure 2.**
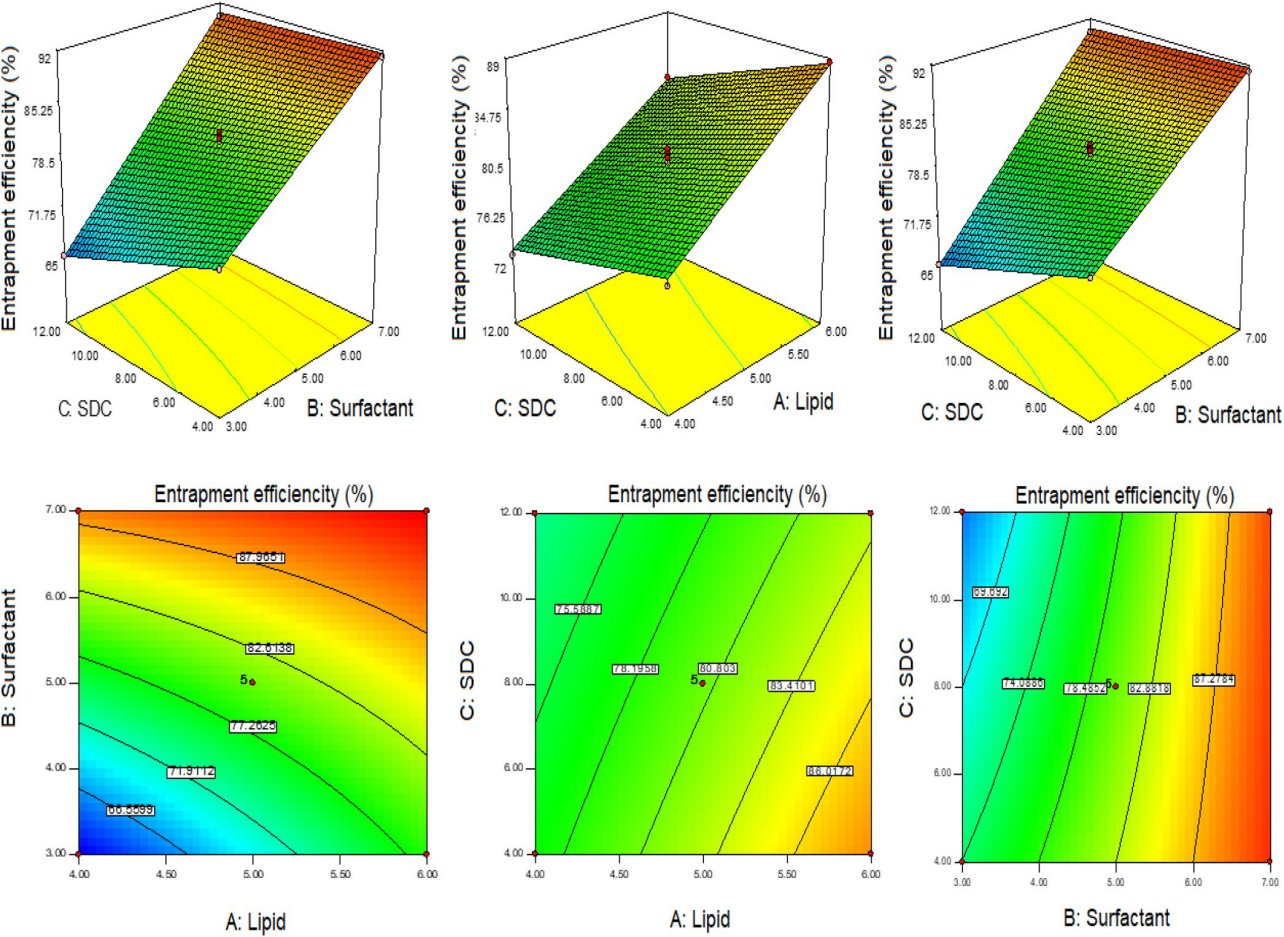
3D and contour plots showing the effect of independent variables on entrapment efficiency of QT-BS.

**Figure 3.**
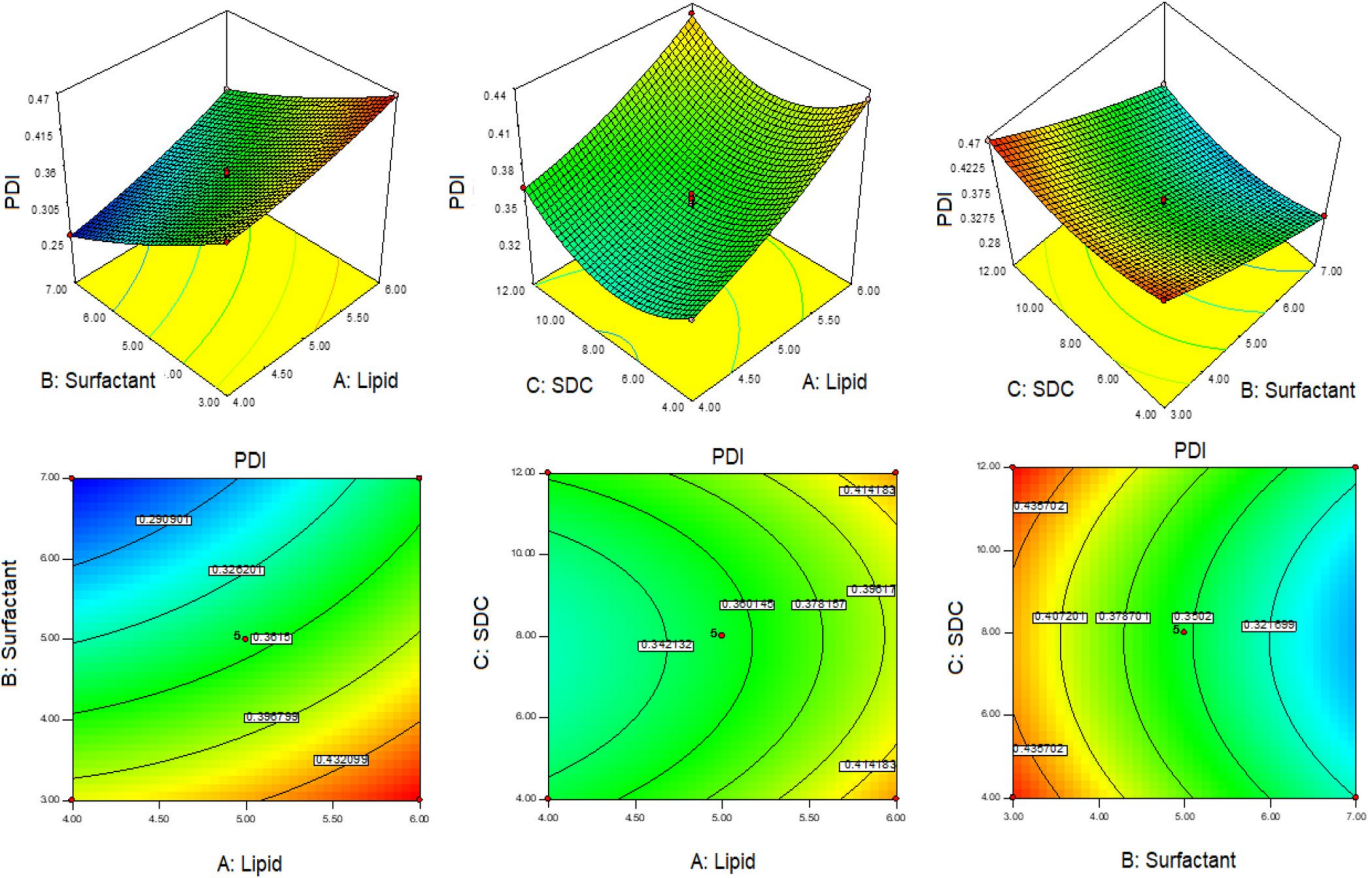
3D and contour plots showing the effect of independent variables on PDI of QT-BS.

The optimization of QT-BS was done by Box-Behnken statistical design. The independent parameter was selected from the preliminary study (data not shown). The thirteen formulations were obtained in different compositions and the values of their responses were determined and are depicted in Table 1. The data for VS, EE, and PDI were fitted into various experimental models (linear, second-order, and quadratic), and it was found that the quadratic model was the best-fitted model for VS, PDI, and 2nd order for EE. The statistical analysis (R^2^, predicted R^2^, adjusted R^2^, standard deviation, and PREES) of all applied models were calculated and depicted in Table 2. The R^2^ of the best-fitted model was greater than that of other models (Table 2). The PREES value of quadratic for VS, PDI, and 2nd order for EE was found to be less than the other models, revealing them as the best-fitted model. The predicted R^2^ is a judicious agreement with the adjusted R^2^ of all fitted models for each response (Table 2) as well as adequate precision, which was greater than 4 for the best-fitted model for each response, indicating that the model was well fitted and could be used to navigate the design space. The ANOVA of the best-fitted model for each response was calculated, and the results are given in Table 3. The lack of fit of the fitted model for each response was found to be non-significant (*P* > 0.05) revealing that the model was well fitted. The response plot (3D and contour) was created for all best-fitted models to explain the effect of the independent variable on the response (Figures 1–3). However, the actual and predicted values of each response of the best-fitted model were also constructed, explaining the closeness between actual and predicted values.

**Table 2.** Statistical summary result of applied model on vesicle size, EE and PDI.

Vesicle size						
Source	Std. Dev.	R^2^	Adjusted R^2^	Predicted R^2^	PRESS	Remark
Linear	17.21	0.9207	0.9024	0.8449	7532.77	–
2FI	10.36	0.9778	0.9646	0.9315	3326.78	–
Quadratic	1.51	0.9996	0.9992	0.9990	44.92	Suggested
**EE**						
Source	Std. Dev.	R^2^	Adjusted R^2^	Predicted R^2^	PRESS	Remark
Linear	2.15	0.9519	0.9408	0.9009	124.41	–
2FI	0.62	0.9968	0.9949	0.9901	12.36	Suggested
Quadratic	0.53	0.9984	0.9964	0.9828	21.53	–
**PDI**						
Source	Std. Dev.	R^2^	Adjusted R^2^	Predicted R^2^	PRESS	Remark
Linear	0.02	0.8983	0.8748	0.834015	0.0089	–
2FI	0.02	0.9050	0.8480	0.706717	0.0158	–
Quadratic	0.003	0.9992	0.9982	0.997411	0.0001	Suggested

**Table 3.** ANOVA resulted of optimized selected model for all responses (VS, EE and PDI).

Source	Vesicle size				Entrapment efficiency				PDI			
	Sum of squares	DF	F value	*p*-value Prob > F	Sum of squares	DF	F value	*p*-value Prob > F	Sum of squares	DF	F value	*p*-value Prob > F
Model	48,559.88	9	2336.84	<0.0001	1251.74	6	531.43	<0.0001	0.054	9	1038.442	<0.0001
A-lipid	11,320.42	1	4902.94	<0.0001	228.35	1	581.70	<0.0001	0.01	1	1965.97	<0.0001
B-surfactant	32,432.86	1	14,046.88	<0.0001	917.83	1	2338.03	<0.0001	0.037	1	6423.51	<0.0001
C-SDC	972.12	1	421.035	<0.0001	49.148	1	125.19	<0.0001	0.00007	1	12.63	0.009
AB	702.25	1	304.14	<0.0001	40.52	1	103.22	<0.0001	0.0003	1	52.89	0.0002
AC	49	1	21.22	0.0025	0.51	1	1.30	0.28	0.00005	1	9.72	0.016
BC	2025	1	877.04	<0.0001	15.37	1	39.15	<0.0001	0	1	0	1.0000
A^2^	440.21	1	190.65	<0.0001	–	–	–	–	0.0003	1	56.32	0.0001
B^2^	0.21	1	0.092	0.7701	–	–	–	–	0.0003	1	47.13	0.0002
C^2^	554.42	1	240.12	<0.0001	–	–	–	–	0.004	1	723.94	<0.0001
Residual	16.16	7	–	–	3.92	10	–	–	0.00004	7		
Lack of fit	1.36	3	0.12	0.9419	3.23	6	3.10	0.14	0.000005	3	0.20	0.89
Pure error	14.81	4	–	–	0.69	4	–	–	0.00003	4	–	–
Cor total	48,576.04	16	–	–	1255.66	16	–	–	0.054147	16	–	–

### Effect of lipid, span-60, and SDC on vesicle size

The quadratic model was found to be the best fit model for VS. The polynomial equation was given below:

(12) $$\text{Vesicle}\;\text{size}\;\left(Y1\right)\;=+220.80+37.62A-63.67B+11.02C-13.25\text{AB}-3.50\text{AC}-22.50\text{BC}+10.{22A}^{2}+\;0.{22B}^{2}+\;11.{48\;C}^{2}$$

**Figure 4.**
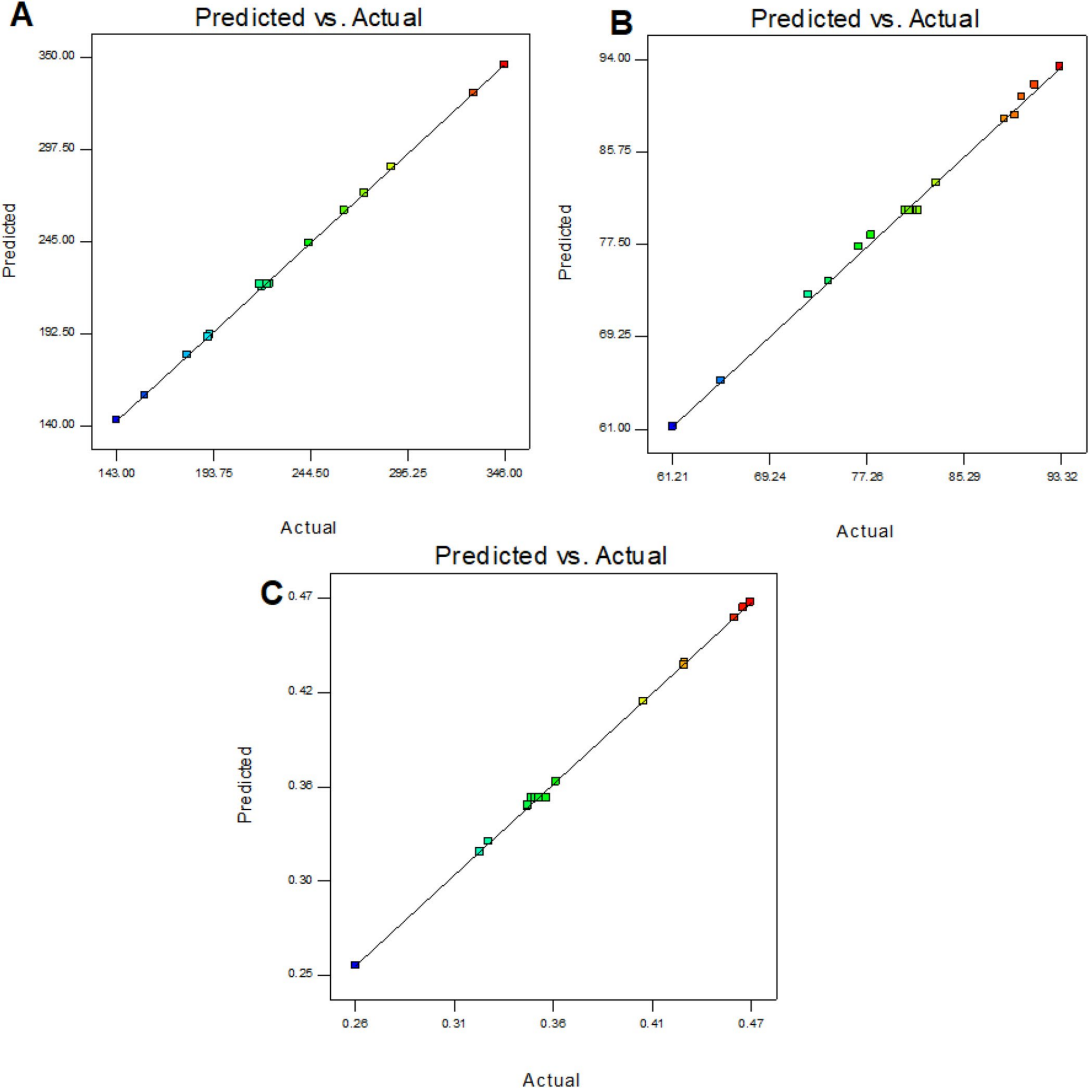
Graphical relationship of the actual and predicted value of the VS (A), EE (B), and PDI.

In this polynomial equation, the positive and negative signs represented the favorable and unfavorable effects on VS. The model terms A, B, C, AB, AC, BC, A^2^, and C^2^ exhibited a significant effect (*P* < .05) whereas B^2^ showed an insignificant (*P* > 0.05) effect on VS. Table 2 shows the results of fitting VS data into various experimental models such as linear, 2nd order, and quadratic. The quadratic model was significantly (*P* < .0001) fitted than the other models because it had the highest R^2^ (0.9996). The PRESS value of the quadratic model was significantly less (44.92) than the other models (Table 2). The ANOVA of the quadratic model was calculated, and the result showed the significant and nonsignificant effects of the independent variable over the VS (Table 3). The lack of fit was found to be insignificant (*P* > 0.05), which gave favor to the fitted quadratic model. The adequate precession was > 4 (173.82), representing a satisfactory signal to the fitted model. The predicted R^2^ (0.9991) was in realistic agreement with the adjusted R^2^ (0.9992). The response surface plot (3D &contour) was plotted and depicted in Figure 1. The relationship between the actual and predicted value of the VS is expressed graphically and is depicted in Figure 4(A). The VS of all QT-BS formulations was found in the range of 143.51 nm (QT-BS3) to 346.34 nm (QT-BS3). Increasing the lipid (A, %) concentration increases the VS size because it enhances the viscosity of the solution, which leads to a slow diffusion rate of the organic phase to the external phase (aqueous phase) (Pradhan et al., 2008) and increased lipid bilayer thickness BS. In addition, increased lipid content and the VS decreased due to incomplete emulsification (insufficient surfactant). A similar type of observation was found in bupivacaine liposome (Lu et al., 2021), and BS delivery of diclofenac (Salem et al., 2022). The second factor, i.e. Span-60 (B, %), exhibited a negative effect on VS. By increasing the concentration of Span-60, the VS decreased because it reduced the interfacial tension (energy) between the lipid and aqueous phase. A similar type of observation was found in BS delivery of dapsone (El-Nabarawi et al., 2020) and nanovesicles delivery of felodipine (Yusuf et al., 2014). The third factor was SDC (C, %), which exhibited a negative effect on the VS of BS. On increasing the concentration of SDC, the surface negative charge increases, thereby the VS of BS increases (El Zaafarany et al., 2010). However, the VS of BS also increased with increasing SDC concentration due to the bulkiness property of SDC (Abdelbary et al., 2016).

### Effect of lipid, span-60, and SDC on EE

The polynomial equation of EE was constructed, which explained the effect of the formulation variable over the response.

(13) Entrapment efficiency (Y2, EE) =+80.45+ 5.34 A+ 10.71B−2.48C−3.18AB−0.36 AC+ 1.96 BC

The model terms A, B, C, AB, and BC exhibited a significant effect (*P* < .05), whereas AC showed an insignificant (*P* > 0.05) effect on EE. The statistical analysis of EE was applied in the linear, second order, and quadratic, and the results are given in Table 2. The 2nd order (2F1) model was significantly (*P* < .0001) fitted to EE than other models because R^2^ was found to be high (0.9969). The PRESS value of the quadratic model was significantly less (12.37) than the other models (Table 2). The ANOVA of the 2nd order model was calculated, and the result showed the significant and nonsignificant effects of the independent variable over the EE (Table 3). The lack of fit was found to be insignificant (*P* > 0.05), which is favorable to the fitted 2nd order (2F1) model. The adequate precession was > 4 (79.86) and represented a satisfactory signal to the fitted model. The predicted R^2^ (0.9902) was in realistic agreement with the adjusted R^2^ (0.9950). The response surface plot (3D & contour) of 2nd order (2F1) was plotted and depicted in Figure 2. The relationship between the actual and predicted value of the EE is expressed graphically and is depicted in Figure 4(B). The model terms A (lipid), and B (Span-60) showed a positive, and C (SDC) exhibited a negative effect on EE. The EE of all QT-BS was found in a range of 61.24% (QT-BS1) to 93.21% (QT-BS4). On increasing the concentration of lipid, the EE increased because the viscosity of the organic phase solution increased and prevented the leaching of QT in addition to the aqueous phase. In addition, EE also increased because of increased hydrophobicity as well as due to a longer acyl length chain. A similar type of finding was reported in the glibenclamide-loaded liposome (Maritim et al., 2021). By increasing the Span-60 concentration, the EE efficiency increases because it decreases the interfacial energy as well as raises the viscosity of the dispersion, which is prime to avoid the leakage of QT from BS. In addition, Span-60 has a longer alkyl chain (C18), which proved the high stability of the lipid bilayer and increased the EE by preventing the leakage of the drug (Balakrishnan et al., 2009; Arzani et al., 2015). In the case of the third factor, on increasing the SDC concentration, the EE efficiency was decreased, but this effect was less prominent than lipid and span-60. The decreased EE might be due to the formation of micelles which increase the solubility of the drug, resulting in its release into the external aqueous phase. In addition, the SDC increases the fluidization and flexibility of the lipid bilayer, which increases the leaching drug, thereby decreasing the EE. A similar type of observation was reported in BS delivery of protein and peptide (Aburahma, 2016) and BS-loaded insulin oral delivery (Niu et al., 2014).

### Effect of lipid, span-60, and SDC on PDI

The polynomial equation of PDI was constructed, which explained the effect of the formulation variable over the response (Y3).

(14) $$\text{PDI}\;\left(Y3\right)\;=\;0.353+\;0.038A-\;0.068B+\;0.003C+\;0.009\text{AB}-\;0.004\text{AC}+\;0\text{BC}+\;0.00{9A}^{2}+\;0.00{8B}^{2}+\;0.0{31C}^{2}$$

The model terms A, B, C, AB, AC, A^2^, B^2^, and C^2^ exhibited a significant effect (*P* < .05), whereas BC showed an insignificant (*P* > 0.05) effect on PDI. The statistical analysis of PDI was applied in linear, 2nd order, and quadratic, and the results are given in Table 2. The quadratic model was significantly (*P* < .0001) fitted to PDI than other models because R2 was found to be high (0.9992). The PRESS value of the quadratic model was significantly less (0.0001) than the other models (Table 2). The ANOVA of the quadratic model was calculated, and the result showed the significant and nonsignificant effects of the independent variable over the PDI (Table 3). The lack of fit was found to be insignificant (*P* = .89, F = 0.20), which favors the fitted model. Adequate precession is > 4 (114.77), representing a satisfactory signal to the fitted model. The predicted R^2^ (0.9902) is in realistic agreement with the adjusted R^2^ (0.9950). The response surface plot (3D & contour) of the quadratic was plotted and depicted in Figure 3. The relationship between actual and predicted values of the PDI is expressed graphically and is depicted in Figure 4(C). The model terms A (lipid), and C (SDC) showed a positive effect, while B (span-60) exhibited a negative effect on PDI. The PDI of all QT-BS formulations was found in a range of 0.256 (QT-BS2) to 0.467 (QT-BS3). On increasing the concentration of lipid, the PDI increased because the vesicle size of BS increased. A similar type of finding was reported in the glibenclamide-loaded liposome (Maritim et al., 2021). On increasing the span-60 concentration, the PDI was decreased because of the decreased vesicle size. This might be due to a decrease in interfacial energy. In addition, Span-60 has a longer alkyl chain (C18), which proves the high stability of the lipid bilayer and decreases PDI (Khan et al., 2015). In the case of the third variable, i.e. SDC, on increasing the concentration of SDC, the PDI increased due to the increased VS (El Zaafarany et al., 2010).

### Section of optimized QT-BS

The optimized QT-BS formulation was selected on the basis of small VS, low PDI, and optimum EE. The QT-BS3 formulation was selected as an optimized formulation (QT-BS3opt). The composition of QT-BS3opt was 4% lipid, 7% Span-60, and 8% SDC. The actual value of QT-BS3opt had a VS of 143.51 nm, an EE of 89.52%, and a PDI of 0.256. The predicted value of QT-BS3opt obtained from point prediction software was 145.32 nm of VS, 88.99% of EE, and 0.258 of PDI, respectively. It was observed that a very small percentage of the variation between the actual and predicted value of the responses. The QT-BS3opt formulation was used for further analysis.

### Vesicle size, PDI, and zeta potential of optimized QT-BS

**Figure 5.**
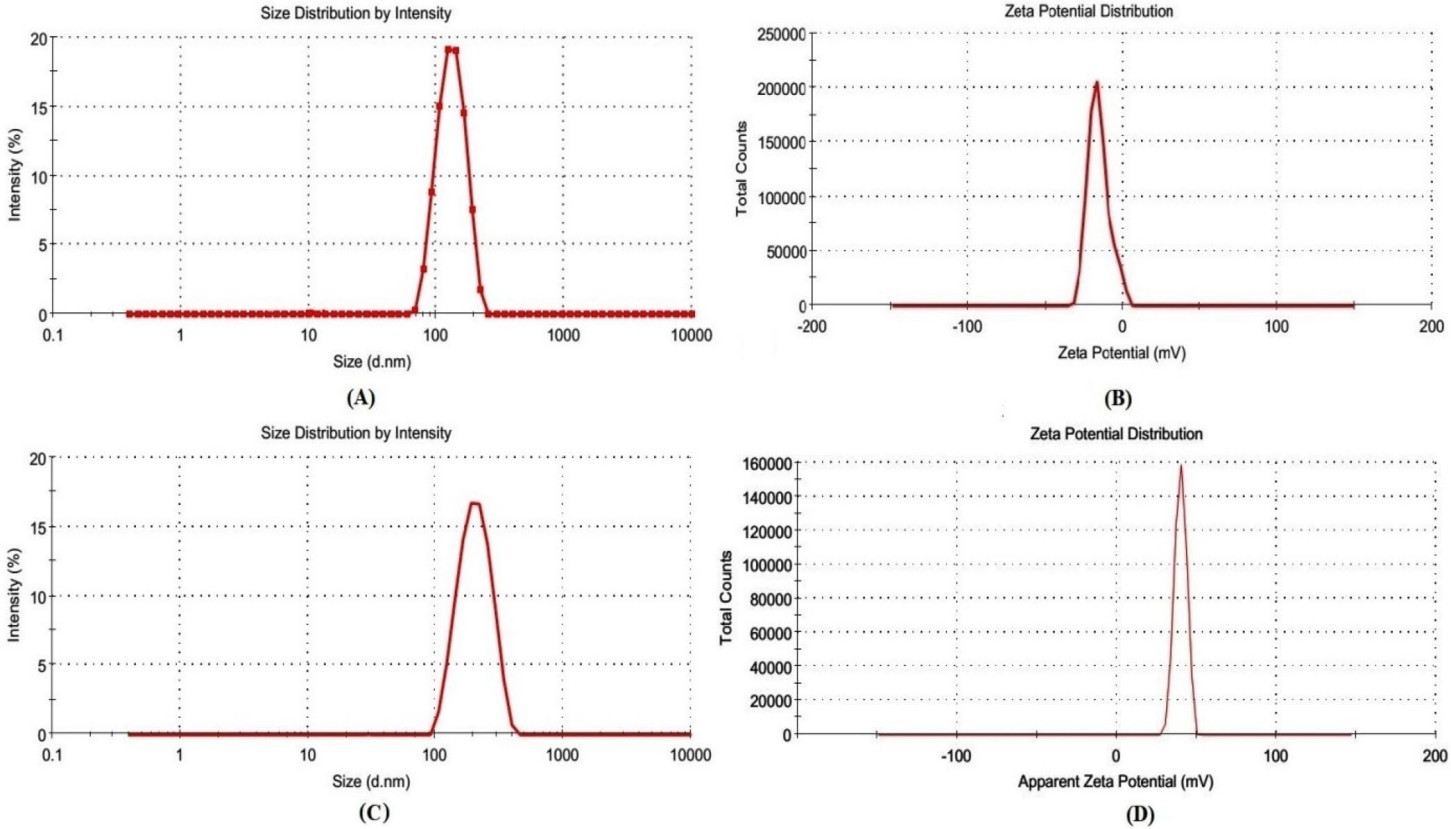
(A) VS, and (B) zeta potential graph of optimized QT-BS formulation (QT-BS3opt) and (C) VS, and (D) zeta potential graph of optimized chitosan coated QT-BS formulation (CS-QT-BS3opt1).

The optimized QT-BS (QT-BS3opt) has a VS of 143.51 nm (Figure 5A), PDI of 0.256, and zeta potential of −15.4 mV (Figure 5B), respectively. The QT-BS3opt was coated with bio-adhesive and biocompatible CS polymer and evaluated for various parameters.

### Development of chitosan-coated QT-BS

The QT-BS3opt was successfully coated with natural bio-adhesive chitosan (CS) polymer. The different CS-coated QT-BS formulations are given in Table 4.

**Table 4.** Composition of CS-QT-BS and their vesicle size and PDI.

Formulation code	CS concentration (%)	Vesicle size (nm)	PDI
CS-QT-BS3opt	–	143.51 ± 3.12	0.256 ± 0.002
CS-QT-BS3opt1	0.20	180.32 ± 5.27	0.282 ± 0.004
CS-QT-BS3opt2	0.25	223.72 ± 4.65	0.320 ± 0.005
CS-QT-BS3opt3	0.30	253.54 ± 5.02	0.357 ± 0.006

### Characterization chitosan-coated QT-BS

The VS and PDI of various CS-coated formulations (CS-QT-BS3opt1-CS-QT-BS3opt3) were analyzed by using a zeta-sizer and the result is depicted in Table 4. It was observed that by increasing the concentration of CS, the VS and PDI were increased (180.32 ± 5.27 nm to 253.54 ± 5.02 nm, at 0.20 to 0.30% CS). It is due to the coating of CS on the surface of BS. The CS-QT-BS3opt1 was chosen as an optimized CS-coated QT-BS formulation with a VS of 180.32 ± 5.27 nm (Figure 5C), a PDI of 0.282 ± 0.004, and zeta potential of +35 ± 2 mV (Figure 5D) based on a minimum VS and PDI.

### X-ray diffraction analysis

**Figure 6.**
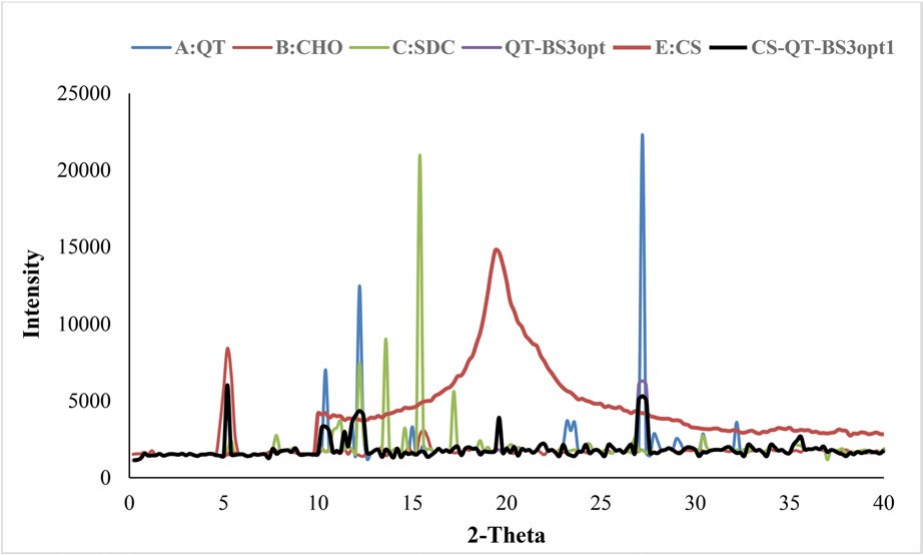
XRD curve of (A) QT, (B) CHO, (C) SDC, (D), QT-BS3opt, (E) CS and (F) CS-QT-BS3opt1 formulation.

The X-ray diffraction spectra of QT, CHO, SDC, QT-BS3opt, CS, and CS-QT-BS3opt1 were recorded at the 2-theta level, and the data is expressed in Figure 6. The QT spectra exhibited a characteristic peak at 10.4^○^, 12.2^○^, and 27.2^○^, respectively (Figure 6A) indicating crystalline characteristics of the drug. The CHO showed a peak at 5.2^○^ (Figure 6B) and SDC exhibited characteristic peaks of 12.2^○^, 13.8^○^, 15.4^○^, and 17.2^○^, respectively (Figure 6C). The QT-BS3opt spectra showed less intense peaks of QT, indicating (Figure 6D) that the drug was encapsulated into the BS matrix. The CS showed the characteristic broad peak at 19.4^○^ (Figure 6E). In spectra of the CS-QT-BS3opt1 formulation, the QT peak intensity was further reduced (Figure 6F) than QT-BS3opt. This might be due to the coating of CS on QT-BS. It revealed that QT was encapsulated in the polymer and lipid matrix. A similar type of finding was reported in curcumin-loaded BS (Liu et al., 2015).

### In-vitro release study

**Figure 7.**
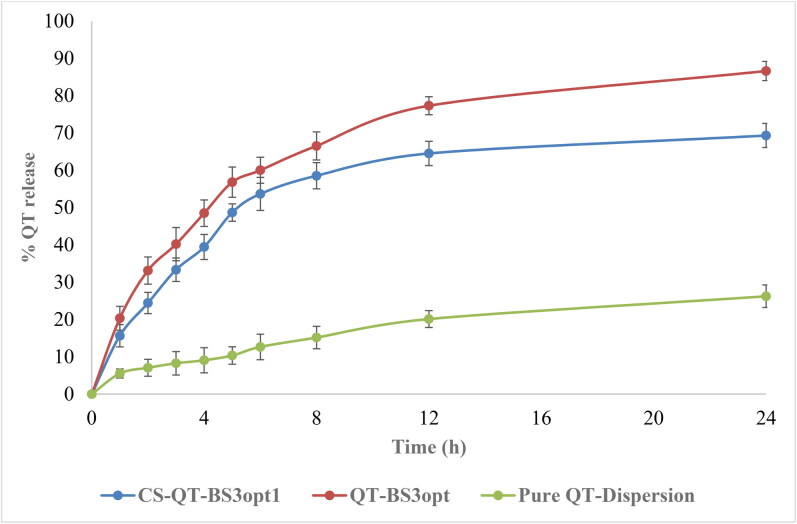
*In-vitro* release profile of CS-QT-BS3opt1, QT-BS3opt, and pure QT-dispersion. The values are expressed as mean ± SD, *n* = 3.

**Figure 8.**
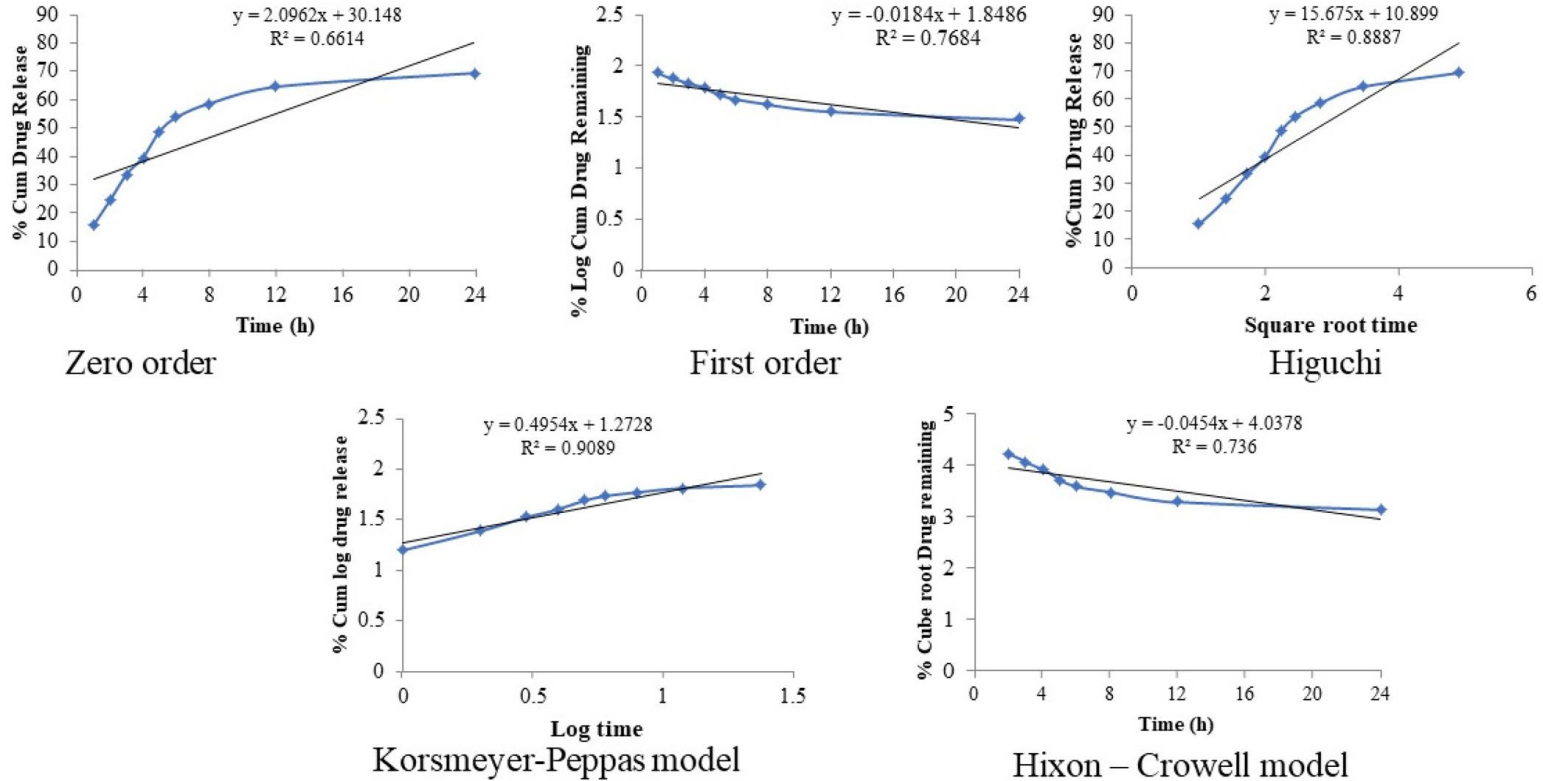
Kinetic models applied to evaluate the best-fitted model in *in-vitro* release profile of optimized QT-BS formulation.

The release of QT from pure QT-dispersion, QT-BS3opt, and CS-QT-BS3opt1 was determined by using the dialysis bag technique, and the result is depicted in Figure 7. The QT releases from pure QT, QT-BS3opt, and CS-QT-BS3opt1 dispersion were found to be 26.23 ± 3.03%, 86.62 ± 3.23%, and 69.32 ± 2.57%, respectively. The result showed QT release from QT-BS3opt and CS-QT-BS3opt1 was found to be in a biphasic pattern. Initially, quick releases (33.13% from QT-BS3opt and 24.43% from CS-QT-BS3opt1) in 2 h due to the release of surface drug and later sustained-release (86.62 ± 3.23% and 69.32 ± 2.57%, respectively) up to 24 h due to release from the inner core matrix by diffusion, swelling or erosion of the matrix system (Zhai et al., 2013). The QT-BS3opt exhibited significantly more release of QT in 24 h due to the nanosize of the vesicle and high effective surface area. Due to the high effective surface area, more dissolution medium contact with the formulation leads to increases in the release (Wu et al., 2017). The high release of QT from the formulation was also due to the presence of bile salt, which contributed to the solubilization of QT into the release medium. The pure QT-dispersion exhibited slow release due to poor solubility in aqueous media. However, CS-QT-BS3opt1 exhibited less release than QT-BS3opt because of the presence of the CS layer on BS, which may provide prolonged release of QT in the body (Liu et al., 2015). The release profile of the CS-QT-BS3opt1 formulation was subjected to various kinetic models, i.e. zero order, first order, Higuchi model, Korsmeyer-Peppas, and Hixon-Crowell model, and the result is depicted in Figure 8. The R^2^ values of zero order, first order, Higuchi, Korsmeyer-Peppas, and Hixon–Crowell models were found to be 0.6614, 0.7684, 0.8887, 0.9089, and 0.7360, respectively. The Korsmeyer-Peppas model has a maximum R^2^ (0.9089) and is selected as the best-fitted model. The exponent value is 0.495 and it lies between 0.45<*n* < 0.89, indicating anomalous behavior or non-fickian diffusion of the drug from the nanoformulation matrix.

### Ex-vivo bio-adhesion study

This study was performed using pig mucin because it has a negative charge and is similar to the intestine mucin of humans (negative charge). The percentage of bio-adhesion represented the amount of mucin bound to the formulation. The bio-adhesion of QT-BS3opt and CS-QT-BS3opt1 was calculated and found to be 20.82 ± 1.45% and 76.43 ± 2.42%, respectively. The CS-QT-BS3opt1 exhibited significantly higher bio-adhesion (*P* < .001) than the QT-BS3opt due to the positive charge of CS bound with the negative charge of mucin (Sogias et al., 2008). The cationic charge on CS-QT-BS3opt1 was confirmed by measuring the zeta potential. Furthermore, because of the positive charge of the CS-QT-BS3opt1 formulation, it can easily bind with negatively charged intestinal mucin and maybe increase the formulation’s residence time (Khalifa et al., 2017).

### Ex-vivo permeation study

**Figure 9.**
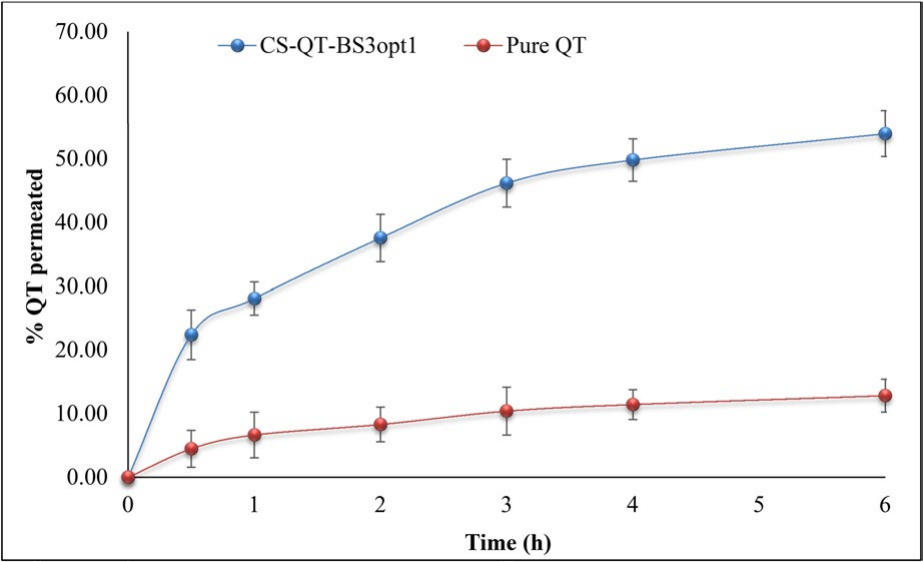
Percentage pure QT permeated from CS-QT-BSopt1 and pure QT-dispersion. The values are expressed as mean ± SD, *n* = 3.

*Ex-vivo* permeation studies of pure QT-dispersion, QT-BS3opt and CS-QT-BS3opt1 were analyzed across the egg membrane. The permeation data is expressed in Figure 9. The percentage of QT permeated from pure QT-dispersion, QT-BS3opt, and CS-QT-BS3opt1 were found to be 12.84 ± 2.59%, 42.5 ± 2.82%, and 53.98 ± 3.59%, respectively. CS-QT-BS3opt1 exhibited significantly (*P* < .05) high flux (permeation), i.e. 4.20-fold more than pure QT dispersion and 1.27-fold higher than QT-BS3opt. The APC of pure QT-dispersion, QT-BS3opt, and CS-QT-BS3opt1 was found to be 2.2 × 10^−3 ^µg/sec, 6.6 × 10^−3 ^µg/sec, and 8.7 × 10^−3 ^µg/sec respectively. The higher permeation of QT-BS3opt1 than pure QT is due to the presence of bile salt (SDC), which enhanced the fluidity of the membrane (Shanmugam et al., 2009). However, the CS-QT-BS3opt1 showed significantly higher permeation than the QT-BS3opt due to the positive charge on the BS surface. The positive charge interacts with the negative charge of the membrane and enhances the permeation (Sohail et al., 2016).

### In-vitro anti-oxidant

#### DPPH radical scavenging method

**Figure 10.**
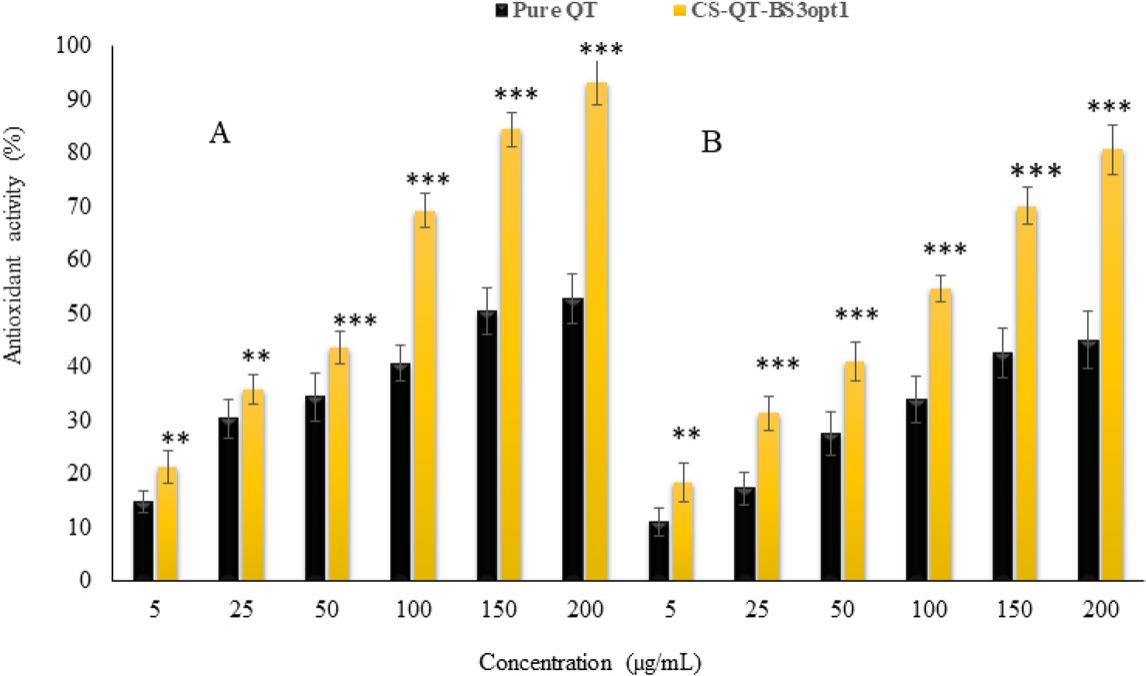
Antioxidant activity of pure QT and CS-QT-BS3opt1 by DPPH radical scavenging (A) and ABTS scavenging method (B). the values are expressed as mean ± SD, *n* = 3. ** and *** indicated that CS-QT-BS3opt1 is significantly different activity at *P* < .01 and *P* < .001 than pure QT.

Figure 10(A) shows the antioxidant potential of pure QT-dispersion and CS-QT-BS3opt1 by the DPPH method. It was observed that the antioxidant potential of QT and CS-QT-BS3opt1 depended upon the concentration of QT. The antioxidant potential of QT from pure QT-dispersion is 14.84%–52.84% antioxidant activity at 5–200 µg/ml concentration. However, CS-QT-BS3opt1 showed antioxidant activity of 21.43%–93.24% at 5–200 µg/ml concentration. The result showed that CS-QT-BS3opt1 exhibited significantly higher (*P* < .05) antioxidant potential at all concentrations than pure QT. The maximum activity of CS-QT-BS3opt1 was 93.24 ± 4.65% at 200 µg/ml than pure QT (52.84 ± 4.12% at 200 µg/ml). The high activity of QT in CS-QT-BS3opt1 may be due to the increased solubility of QT because of the presence of bile salt and CS.

#### ABTS scavenging method

The result of the antioxidant activity of pure QT and CS-QT-BS3opt1 by the ABTS scavenging method is depicted in Figure 10(B). The data showed the antioxidant activity of QT from both formulations was dependent upon concentration. The antioxidant activity of pure QT was 11.05%–45.10% at 5–200 µg/ml. However, the antioxidant activity of QT in the form of CS-QT-BS3opt1 formulation was 18.43%–80.76% at 5–200 µg/ml. The result showed that QT from CS-QT-BS3opt1 exhibited remarkably (*P* < .05) higher activity at each concentration than pure QT. The QT showed maximum activity in pure form was 45.10 ± 3.34% at 200 µg/ml and in the form of CS-QT-BS3opt1 80.76 ± 4.05% at 200 µg/ml. The CS-QT-BS3opt1 showed higher antioxidant activity than pure QT due to an increase in the solubility of QT in the nano-system. The result showed that antioxidant activity by the ABTS method is less than that by the DPPH method.

#### Cytotoxicity study

**Figure 11.**
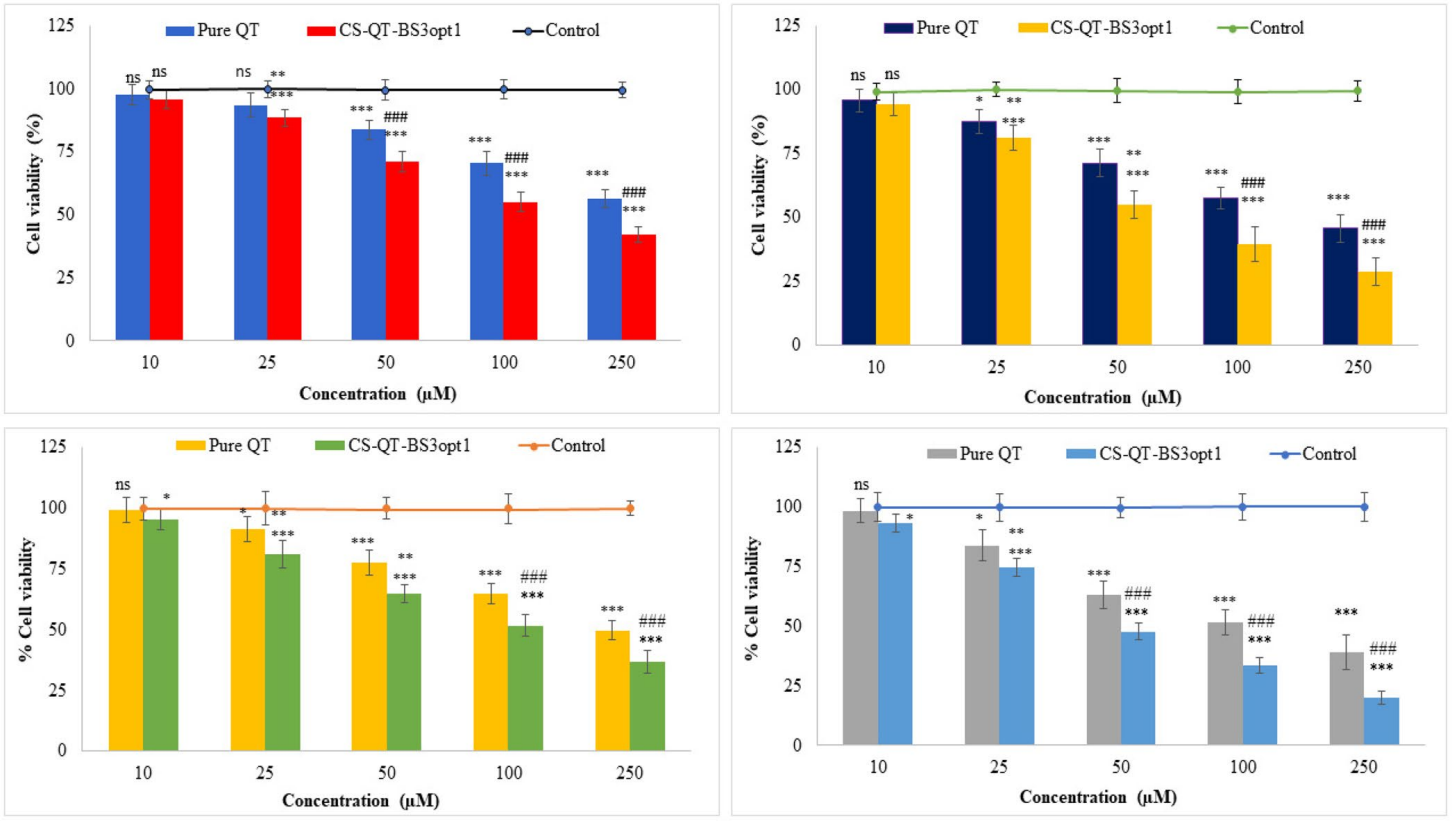
Cell viability profiles of pure QT and CS-QT-BS3opt1against: (A) MDA-MB-231 cells after 24 h; (B) MDA-MB-231 cells after 48 h; (C) MFC7 cells after 24 h; (D) MFC7 cells after 48 h. Tukey Kramer test was used to compare different groups. * and *** significant and highly significant compared to control, ** and ### significant and highly significant as compared to pure QT, ns—non-significant.

The cytotoxicity study of CS-QT-BS3opt1 and pure QT dispersion was performed in MDA-MB-231 and MFC-7 cell lines and the results are depicted in Figure 11. The CS-QT-BS3opt1 exhibited a significant death of breast cancer cells (MFC7 and MDA-MB-231 cells) at 10–250 µM in 24 and 48 h compared to control and pure QT. Pure QT demonstrated cell viability at concentrations of 10 µM (97.72%), 25 µM (93.56%), 50 µM (83.68%), 100 µM (70.43%), and 250 µM (56.39%) at 24 h and 10 µM (95.67%), 25 µM (83.43%), 50 µM (71.21%), 100 µM (57.38%), and 250 µM (45.62%) after 48 h against MDA-MB-231 cell line. However, the CS-QT-BS3opt1 exhibited significant (*P* < .05) activity against the MDA-MB-231 cell line at different concentrations, i.e. 10 µM (95.65%), 25 µM (88.51%), 50 µM (71.25%), 100 µM (55.02%), and 250 µM (42.12%) in 24 h, and 10 µM (94.23%), 25 µM (81.14%), 50 µM (54.82%), 100 µM (39.43%), and 250 µM (31.65%) in 48 h. The pure QT showed activity against the MFC7 cell line at different concentrations of 10 µM (99.24%), 25 µM (91.35%), 50 µM (77.43%), 100 µM (64.65%) and 250 µM (49.73%) in 24 h and 10 µM (98.23%), 20 µM (87.56%), 50 µM (63.02%), 100 µM (51.43%) and 250 µM (38.76%) in 48 h. However, CS-QT-BS3opt1 exhibited significant activity, i.e. 10 µM (95.34%), 25 µM (81.23%), 50 µM (61.65%), 100 µM (51.58%) and 250 µM (36.76%) in 24 h and 10 µM (93.02%), 25 µM (76.62%), 50 µM (47.7%), 100 µM (33.28%) and 250 µM (19.98%) in 48 h against the MFC7 cell line. In both cell lines, CS-QT-BS3opt1 had significantly higher cell cytotoxicity at each concentration than pure QT and control after 24 and 48 h. It revealed that cytotoxicity depends upon the concentration of QT. The maximum activity was found at 250 µM for both samples (QT and CS-QT-BS3opt1) in 24 and 48 h. As compared to pure QT, CS-QT-BS3opt1 showed 1.61-fold cytotoxicity against MFC7 and 1.44-fold cytotoxicity against MDA-MB-231 in 48 h. The IC50 of pure QT was 244.8 µM and 213.10 µM against MDA-MB-231 in 24 h and 48 h, respectively. In addition, the IC_50_ value of pure QT against MFC-7 was 221.60 µM and 115.70 µM at 24 h and 48 h, respectively. On the other hand, IC50 of CS-QT-BS3opt1 against MDA-MB-231 was 173.7 µM and 68.54 and against MFC-7 was 61.89 µM and 43.87 µM at 24 h and 48 h, respectively. CS-QT-BS3opt1 showed a lower IC_50_ concentration than pure QT. The result showed that QT is more cytotoxic against MFC-7 than CS-QT-BS3opt1. The higher activity of QT from CS-QT-BS3opt1 than pure QT might be due to an increase in the solubility owing to the presence of bile salt and Span-60 that were responsible for the accumulation of more QT at the cell site. Further, the coating with CS also helps to improve the anticancer activity by improving the bio-adhesion and opening the tight junction of the cancer cell (Adhikari and Yadav., 2018). CS holds a positive charge due to the amino group, causing the attraction toward the negative charge of cancer cells rather than normal cells and increasing the anticancer activity (Zhang et al., 2010; Wimardhani et al., 2014).

#### Antibacterial activity

**Figure 12.**
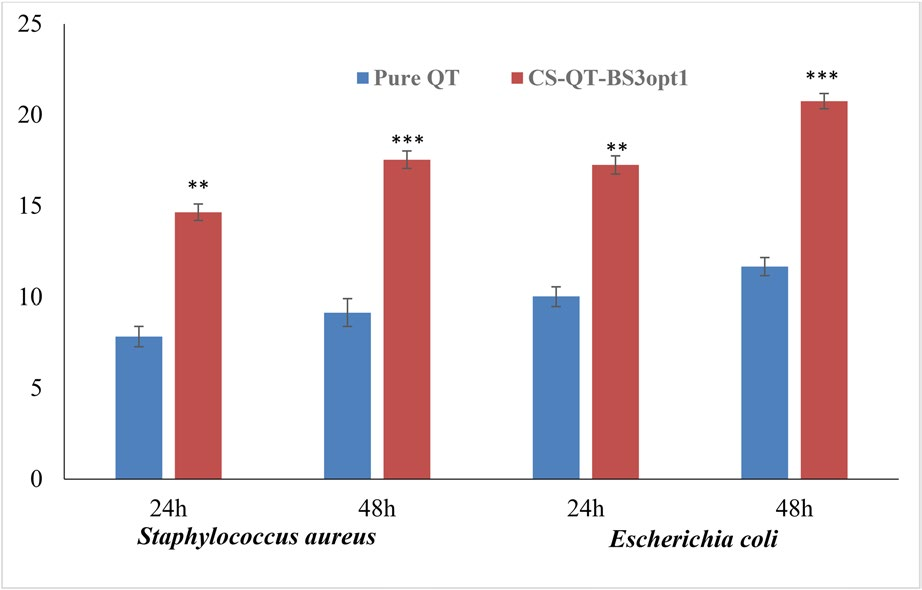
Antimicrobial evaluation of pure QT and CS-QT-BS3opt1 against S*. aureus* (Gram-positive) and *E. coli* (Gram-negative) in 24 and 48 h in the terms of zone of inhibition (ZOI). The values are expressed as mean ± SD, *n* = 3, ** and *** indicating that CS-QT-BS3opt1is significantly different at *p* < .01 and *p* < .001, respectively from pure QT.

The antimicrobial evaluation of pure QT dispersion and CS-QT-BS3opt1 was done on S. aureus (Gram-positive) and E. coli (Gram-negative) microbial strains using the cup plate method up to 48 h, and the results are expressed in Figure 12. The result showed that QT showed more susceptibility to E. coli than S. aureus in the same concentration. The pure QT exhibited a ZOI of 7.84 ± 0.56 mm and 10.02 ± 0.50 mm against S. aureus and E. coli at 24 h, respectively. The ZOI of pure QT was found to be 9.15 ± 0.75 mm and 11.66 ± 0.42 mm against S. aureus and E. coli, respectively, in 48 h. The CS-QT-BS3opt1 formulation exhibited significant (*P* < .05) high antibacterial activity against both tested bacteria at both tested points (24 and 48 h) in comparison to pure QT. The ZOI of QT from CS-QT-BS3opt1 was found to be 14.65 ± 0.45 mm against S. aureus and 17.25 ± 0.50 mm against E. coli. However, CS-QT-BS3opt1 showed significantly more activity in 48 h, i.e. 17.54 ± 0.48 mm against S. aureus and 20.76 ± 0.42 mm against E. coli. The high activity of QT from CS-QT-BS3opt1 is due to the nano size of the vesicle, which provides a higher surface area for diffusion (Abdelbary et al., 2016). The presence of surfactant and bile salt in BS increases the solubility and membrane permeability of the membrane, leading to enhanced activity (Sannasiddappa et al., 2017). The coating with the CS to BS formulation further increases the antibacterial activity of QT because of the sustained release of QT and high permeability (Li et al., 2018). The antibacterial activity of QT is by damaging the cells and cell membranes of gram-positive and gram-negative bacteria (Wang et al., 2018).

## Conclusion

In this work, the QT-loaded bilosome was prepared and optimized by Box-Behnken design. The formulation unveiled a nano-range of vesicle size, high entrapment efficiency, and low PDI. The result of XRD showed the amorphization of QT after incorporation into CH-BLOs. CS-QT-BS3opt1 exhibited sustained drug release up to 24 h with Korsmeyer-Peppas kinetic as a best-fitted model. CS-QT-BS3opt1 displayed better bioadhesion as well as better permeability flux as compared to QT-dispersion. CS-QT-BS3opt1 exhibited a greater antibacterial potential against E. coli than S. aureus than QT-dispersion. As compared to pure QT-dispersion, CS-QT-BS3opt1 showed 1.61-fold cytotoxicity against MFC7 and 1.44-fold cytotoxicity against MDA-MB-231 in 48 h, indicating more QT cytotoxic against MFC-7 than CS-QT-BS3opt1. Finally, all of the findings suggest that CS-QT-BS3opt1 could be a viable technique for the increment of QT efficacy against certain diseases.
